# Cerebral ischemia-reperfusion model in rabbits: relationship between dexmedetomidine and biochemical parameters in lowering intraparenchymal pressure

**DOI:** 10.1186/cc10935

**Published:** 2012-03-20

**Authors:** A Tavlan, ME Ustun, A Yosunkaya, A Ak, A Kiyici, HK Bardakcı, F Gok

**Affiliations:** 1 Selcuk University, Meram Medical Faculty, Konya, Turkey; 2 Farabi Hospital, Konya, Turkey

## Introduction

The effect of dexmedetomidine in two different doses on the levels of endothelin-1 (ET-1) and prostoglandin I_2 _(PGI_2_) in blood and cerebrospinal fluid (CSF) of rabbits via the transient global cerebral ischemia model was studied to determine its intraparenchymal pressure (IPP) reduction mechanism.

## Methods

Twenty-four New Zealand type rabbits were employed and randomly distributed into four groups. Group I (sham group, *n *= 6): craniotomy was performed only. Group II (control group, *n *= 6): bilateral carotid arteries were clamped for 60 minutes after craniotomy, then reperfusion was performed for 60 minutes. In Group III (*n *= 6) and Group IV (*n *= 6), 80 μkg^-1 ^and 320 μkg^-1 ^dexmedetomidine was administered within the first 10 minutes of the reperfusion procedure respectively. Blood and CSF samples were collected 120 minutes after craniotomy. Mean arterial pressures (MAP), heart rates (HR), IPP and temperature values were recorded.

## Results

There was no significant difference in MAP values between groups (*P *≥ 0.05). A decrease of HR in Group IV was significantly lower after reperfusion (*P *< 0.05). IPP values after the reperfusion in Groups II and IV were significantly higher than Group I (*P *< 0.05), but no significant increase in Group III (*P *≥ 0.05). ET-1 levels of both blood and CSF were increased in the group with performed ischemia and reperfusion and no treatment (Group II) and the group administered high-dose dexmedetomidine (Group IV) (*P *< 0.05), while the group administered low-dose dexmedetomidine (Group III) was similar to the sham group (*P *≥ 0.05). However, PGI_2 _levels of CSF were significantly decreased in the group administered low-dose dexmedetomidine (*P *< 0.05).

## Conclusion

Dexmedetomidine could decrease intraparenchymal pressure in the transient global cerebral ischemia model when administered at low doses [1,2]. It probably contributed to this reduction by preventing an increase of endothelin levels in blood and CSF as well as decreasing PGI_2 _levels in CSF.
